# *In Vitro* Antioxidant, Antiproliferative, and Phytochemical Study in Different Extracts of *Nyctanthes arbortristis* Flowers

**DOI:** 10.1155/2014/291271

**Published:** 2014-05-20

**Authors:** Manjulatha Khanapur, Ravi K. Avadhanula, Oruganti H. Setty

**Affiliations:** Department of Biochemistry, School of Life Sciences, University of Hyderabad, Professor C. R. Rao Road, Gachibowli, Hyderabad, Andhra Pradesh 500 046, India

## Abstract

*Nyctanthes arbortristis* L. (Oleaceae) is widely used in the Indian system of traditional medicine and is reported to have various biological activities. The present study was intended to evaluate the antioxidant and antiproliferative activities of flower extracts of *Nyctanthes arbortristis*. The shade dried flowers were extracted with 95% ethanol under sonication and the antioxidant activities were investigated using *in vitro* assays along with the determination of phytochemical constituents (total polyphenol and total flavonoid). Arborside C and **β**-monogentiobioside ester of **α**-Crocetin were identified in crude active extracts through LCMS/MS analysis. The antiproliferative activity was carried out by MTT assay by employing different human cancer cell lines. The lowest IC_50_ value of 24.56 ± 6.63 **μ**g/mL was observed against Colo 205 cell line. The extract exhibited significant antioxidant and antiproliferative properties and the observed biological activities in this study provide scientific validation of ethnomedicinal use of this plant.

## 1. Introduction

Cancer is responsible for 12% of the world's mortality and the second-leading cause of death in the world [1]. In spite of much progress in the recent past in the cancer treatments, a key problem in tumor therapy with established cytostatic compounds is the development of drug resistance and acute side effects. Most available drugs suffer from insufficient specificity toward tumor cells [2]. Hence, the identification of better antitumor drugs is the need of the hour. Over the last two decades, number of studies has investigated the diverse health benefits and protective effects of natural substances present in the plants, particularly having antioxidant and antiproliferative properties. The scientific evaluation of medicinal plants used in the preparation of folk remedies has provided modern medicine with effective pharmaceutical drugs for the treatment of many infectious and chronic diseases including cancer [3]. Between 1983 and 1994, more than 60% of the approved anticancer drugs in the United States of America were from natural origin. Most of the anticancer agents have been shown to possess antioxidant potential that can play an important role in the protection of some forms of cancer [4, 5].

Plants as master chemists accumulate a wealth of intricate secondary metabolites, which further deliver effective treatments for a plethora of human diseases. Alternative or complementary traditional therapies are used to cure many diseases potentially that are plant derived or other natural sources. In traditional system of medication the importance to be considered is its broad range of outcomes which often treat the whole person rather than a specific symptom or disease. These therapies also make accessibility to the population living in diverse environmental conditions such as geographical and climatic. Thus make the accessibility to the person(s) in need with less efforts and dependence on other resources which in turn could be economically beneficial as well [6]. The phytochemicals found in plant-based foods also possess biological properties along with their antioxidant property. Consuming of antioxidant-rich foods has several health benefits that help to prevent many diseases [7, 8]. The secondary metabolites such as alkaloids, flavonoids, coumarins, and steroids have been shown to possess antioxidant and anticancer activities in both* in vivo* and* in vitro* models [9–11]. Phytomedicine could be in the form of crude preparations (extracts, tinctures, and essential oils) containing a wide variety of compounds or could be pure molecules with a strong and specific activity. The natural products symbolize safety in contrast to the synthetic drugs, but still there is need to check their efficacy with systematic studies. Therefore, the need for new therapeutic options has prompted many researchers to evaluate the efficacy of compounds found in natural products [12].

*Nyctanthes arbortristis* (Oleaceae) is a mythological plant and possesses high medicinal values in Ayurveda. The popular medicinal uses of* N. arbortristis* include antihelminthic and antipyretic; besides it is used in disorders like rheumatism and skin ailments and as a sedative. Phytochemical investigations of* N. arbortristis* indicated the presence of a large number of phenolic compounds, iridoids, and carotenoids, such as arbortristoside (A, B, C) with many biological activities like anticancer, antileishmania, anti-inflammatory, antiallergic, immunomodulatory, and antiviral [13]. The flowers of this sacred plant have not been explored for antiproliferative activity till date. Therefore, the present study was initiated with the aim of investigating the antioxidant and antiproliferative activities of the flower extracts.

## 2. Materials and Methods

### 2.1. Reagents

Doxorubicin, DPPH, quercetin, and gallic acid were purchased from Sigma Aldrich, USA. Organic solvents and HCl, hexamethylenetetramine, sodium nitrite, aluminum chloride, NaOH, NaCO_3_, and Folin-Ciocalteu reagent were purchased from Merck, India.

### 2.2. Plant Material

The flowers from well grown and healthy plants of* N. arbortristis *were collected in and around the University of Hyderabad, Hyderabad. A voucher specimen (UoH/MDP/NA-00005) has been preserved in our laboratory for future reference.

### 2.3. Preparation of Extract and Phytochemical Screening

The flowers were shade dried and coarsely powdered using electric blender. The powdered material was then extracted with 95% ethanol under sonication. The ethanol solvent was removed under reduced pressure using a rotary vacuum evaporator (Buchii, USA) and dark reddish gummy ethanolic extract of* N. arbortristis* flowers (NafE) was obtained. This ethanolic extract was taken in double distilled water (ddH_2_O) and partitioned with hexane, ethyl acetate, and n-butanol successively and subsequently the organic solvents were recovered under reduced pressure and concentrated. Insoluble part obtained on partitioning between aqueous and organic layers was also collected and concentrated under reduced pressure. Finally, the remaining aqueous part was also concentrated to polar extract by lyophilisation. Thus,* N. arbortristis *ethanolic extract (NafE), hexane extract (NafEHx), ethyl acetate extract (NafEEa), n-butanol extract (NafEBu), insoluble part extract (NafEIn), and aqueous extract (NafEW), total six extracts, were obtained from extraction process and preserved at −20°C for further analysis. Phytochemical screening of secondary metabolites in all six extracts was carried out as described by Harbone [14].

### 2.4. Determination of Phytoconstituents

#### 2.4.1. Determination of Total Phenolic Contents

The amount of total soluble phenolic content in all six extracts (NafE, NafEHx, NafEEa, NafEBu, NafEIn, and NafEW) was determined according to Folin-Ciocalteu method with minor modifications [15]. Briefly, 10 *μ*L of extracts from the stock solution was mixed with 100 *μ*L of Folin-Ciocalteu reagent. After 10 min of incubation at room temperature, 300 *μ*L of 20% Na_2_CO_3_ solution was added and the volume was adjusted to 1 mL using dH_2_O. The mixture was incubated in the dark for 2 h and the absorbance was measured at 765 nm using a UV-Vis spectrophotometer against blank sample. The total phenolic content was measured as gallic acid equivalents (mg GAE)/gm of dry weight (dw) and the values were presented as means of triplicate analysis.

#### 2.4.2. Determination of Flavonoid Contents

Total flavonoid content in extracts was estimated by a colorimetric method as described by Veronica et al. [16] with minor modifications by taking 20 *μ*L of each extract mixed with 500 *μ*L distilled water and 30 *μ*L of 5% NaNO_2_ solution. After 5 min of incubation at room temperature, 60 *μ*L of 10% AlCl_3_ solution was added. Subsequently, 350 *μ*L of 1 M NaOH and 40 *μ*L of distilled water were added to make the final volume of 1 mL. Samples were further incubated for 15 min at room temperature and the absorbance of the samples was measured at 510 nm. The total flavonoids were determined as quercetin equivalents (mg QE)/g of dw and the values were expressed as means of triplicate analysis.

### 2.5. Antioxidant Assays

#### 2.5.1. Total Antioxidant Capacity

The total antioxidant activity of test extracts was evaluated by green phosphomolybdenum complex according to the method of Prieto et al. [17]. An aliquot of 10 *μ*L of extracts was mixed with 1 mL of reagent solution (0.6 M sulphuric acid, 28 mM sodium phosphate, and 4 mM ammonium molybdate) in Eppendorf tubes. Tubes were incubated in a dry thermal bath at 95°C for 90 min. After cooling, the absorbance of the mixture was measured at 695 nm against a blank. Ascorbic acid was used for reference and the reducing capacities of the analyzed extracts were expressed as mg of ascorbic acid equivalents (mg AAE)/g of dw.

#### 2.5.2. DPPH• Radical Scavenging Activity

The hydrogen-donating abilities of extracts were examined according to the method of Cuendet et al. [18] with some modifications using 2, 2-diphenyl-2-picrylhydrazyl hydrate (DPPH) as reagent that offers a convenient and accurate method for titrating the oxidizable groups of natural or synthetic antioxidants. Briefly, 0.004% w/v of DPPH radical solution was prepared in methanol and then 900 *μ*L of this solution was mixed with 100 *μ*L of extract solution containing 20–360 *μ*g/mL of dried extracts. The absorbance was measured at 517 nm after 30 min of incubation. Methanol (95%), DPPH solution, and ascorbic acid were used as blank, control, and reference, respectively. The IC_50_ value represents the concentration of extracts that inhibits 50% of the radical. Scavenging concentration for 50% of DPPH free radical (IC_50_) was calculated from logarithmic regression equation obtained from the values of at least five dilutions of the primary concentration.

### 2.6. Evaluation of Antiproliferative Activity

The six different cell lines that were used in study are colorectal adenocarcinoma (Colo 205); retinoblastoma (Y79); chronic myelogenous leukemia (K562); breast adenocarcinoma (MCF7); breast adenocarcinoma (MDAMB231). The cells lines were obtained from the National Centre for Cell Sciences, Pune, India, and were cultured at a seeding density of 0.2 × 10^6^ in DMEM/RPMI medium supplemented with 10% FBS, 100 U/mL penicillin, and 100 *μ*g/mL streptomycin, respectively, and maintained in a humidified atmosphere with 5% CO_2_ at 37°C. The samples were dissolved in dimethylsulfoxide (DMSO; not exceeding the final concentration of 0.01%) and further diluted in cell culture medium. The antiproliferative response of extract was assessed by MTT assay [19]. Cells (~10,000) were plated in 200 *μ*L growth medium in the presence or absence of the extract (25, 50, 100, and 200 *μ*g/mL) in 96-well culture plates for 24 h. Then the culture plates were centrifuged at 2000 rpm for 10 min at room temperature. 100 *μ*L of supernatant was discarded and 20 *μ*L of MTT (5 mg/mL in PBS) was added to each well and incubated for 4 h at 37°C. The viability of the cells was determined using a spectrophotometer at 570 nm. The IC_50_, that is, the concentration of the extract required to inhibit cell growth by 50%, was determined.

### 2.7. Chromatography Profile: High Performance Liquid Chromatography and Liquid Mass Spectrometry (HPLC/MS/MS)

Agilent 1200 series coupled with DAD-UV detector that was equipped with Agilent Technologies 6520 with Accurate Mass Q-TOF mode was used to perform mass spectrometry and Zorbax SB-C18 column rapid resolution (3.5 *μ*m, 4.6 × 150 mm). The flow rate was 0.45 mL/min, and the injection volume was 3 *μ*L. The analyses were performed using binary gradients of Milli-Q water (with 0.1% formic acid + 10 mM ammonium formate) (solvent A) and HPLC grade acetonitrile (with 0.1% formic acid) (solvent B) with the following elution profile: from 0 min: 35% (B) in (A); 10 min: 55% (B) in (A); 25 min: 95% (B) in (A); 35 min: 35% (B) in (A).

### 2.8. Statistical Analysis

Data were presented as means standard deviation (SD). Statistical analysis was performed using Student's *t*-test analysis and one-way analysis of variance (ANOVA). The results were considered statistically significant when *P* < 0.05. The Dictionary of Natural Products on DVD software (CRC Press, Taylor and Francis Group, https://netbeans.org/) was used to analyze the chromatography profiling data.

## 3. Results

### 3.1. Extraction, Preliminary Phytochemical Screening, Phytoconstituents Assay, and LCMS/MS Analysis 

**Figure 1:**
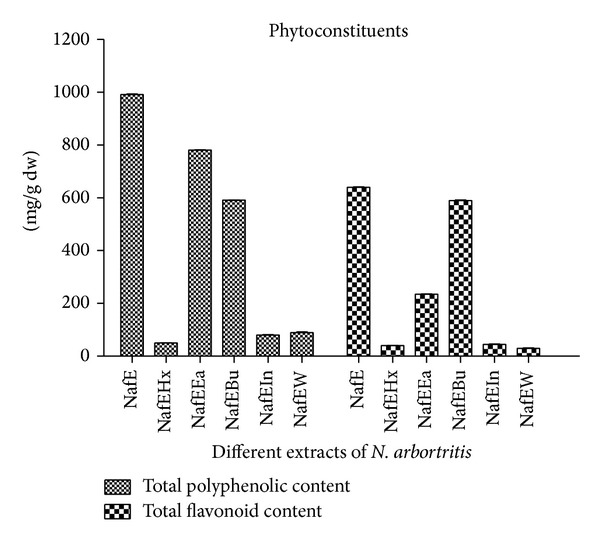
Phytoconstituents content (total flavonoid and total phenolic) in different extracts of* N. arbortristis *flower.

In the present study, the extraction was carried out under ultrasonication using 95% ethanol as the solvent, followed by fractionation of same extract with various solvents with increasing polarity and the final extracts were designated as NafE, NafEHx, NafEEa, NafEBu, NafEIn, and NafEW. These six different extracts were subjected to phytochemical screening to check the presence of different phytoconstituents and results are tabulated in Table 1. The UV profile of NafE, NafEa, and NafBu chromatograms analysed at all wave lengths demonstrated two *λ*_max⁡_ in the region of 240–280 nm and 300–380 nm thus suggesting the presence of flavonoids [20].The results of phytoconstituents in different flower extracts of* N. arbortristis* are presented in Table 1 and Figure 1. The flavonoid content in different extracts (NafE, NafEa, and NafBu) was found to be in the order of 640 ± 2.09 mg QE/100 g; 590 ± 1.09 mg QE/100 g; and 235 ± 1.81 mg QE/100 g, respectively, and by Folin-Ciocalteu method for total phenolic content of NafE, NafEa, and NafBu extracts was shown as 991 ± 0.5 mg GAE/100 g; 781 ± 1.02 mg GAE/100 g; and 591 ± 0.07 mg GAE/100 g, respectively.

**Figure 2:**
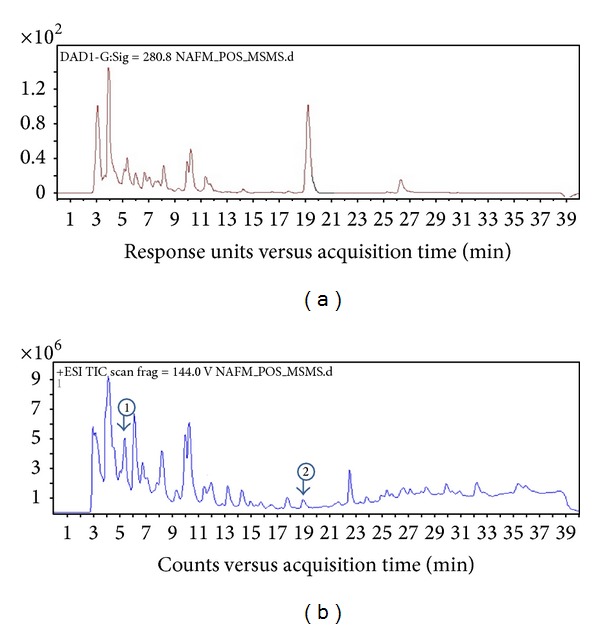
(a) LC-UV chromatogram at 280 nm of ethanolic extract of flowers of* N. arbortristis*. (b) LC-MS total ion chromatogram of the ethanolic extract of flowers of* N. arbortristis*. Compounds: 1 (RT = 5.1) Arborside C and 2 (RT = 19.603) Crocin-3.

**Figure 3:**
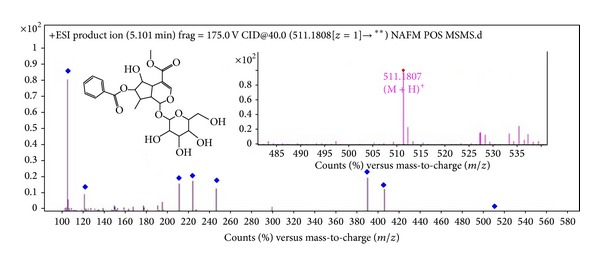
Mass spectrum (TOF MS ES+) of peak number 1 in* N. arbortristis *flowers ethanol extract (identified as Arborside C).

**Figure 4:**
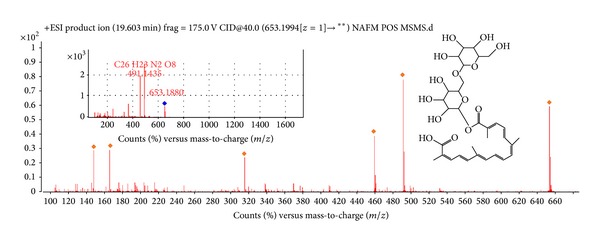
Mass spectrum (TOF MS ES+) of peak number 2 in* N. arbortristis *flowers ethanol extract (identified as Crocin-3).

The NafE was subjected to LC-DAD-ESI-MS to identify the phytochemical(s) by absorption peaks in UV (Figure 2(a)) and with molecular ion in Q-TOF and by their fragmentation (MS/MS) using the positive ionisation mode and observed many peaks (Figures 2(b), 3, and 4); however only two peaks were identified. Peak 1 (Figure 2(b)) (RT = 5.1 min, *λ* = 280 nm, and MW = 510.494) (Figure 3) had [M + H]^+^ at* m/z* 511 and was identified as Arborside C [21], namely, 6 *β*-hydroxyguanine with O-benzoyl substitution with loss of benzoyl that ion appeared at* m/z* 105, benzoate* m/z* 121, and glucoside* m/z* 165 and 6 *β*-hydroxyguanine at* m/z* 244, 228, and 212. Peak 2 (Figure 2(b)) (RT = 20.509 min, *λ* = 440 nm, and MW = 652.27) (Figure 4) had [M + H]^+^ at* m/z* 653.1994 and was identified as carotenoid, glycosides, namely, *β*-monogentiobioside ester of *α*-Crocetin (or Crocin-3) with loss of 1,5-anhydro-D-glucitol that ion appeared at* m/z* 165.0651 and* m/z* 491 carotenoid ester with other fragments at* m/z* 459, 315, and 147 [22]. The reddish-orange coloured tubular calyx of flower of* N. arbortristis *is due to carotenoid pigments (Crocetin and its derivatives) which are reported from the flowers of this plant [22, 23].

### 3.2. Antioxidant and Free Radical Scavenging Ability Assays

**Figure 5:**
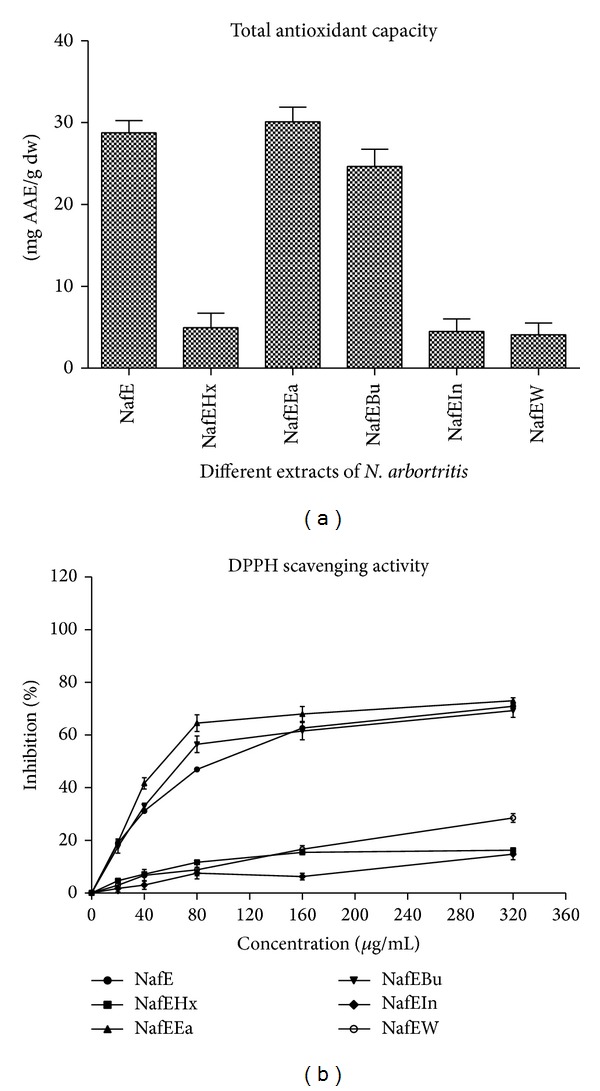
(a) Total antioxidant content in different extracts. All values are expressed as the means ± SEM. (b) DPPH radical scavenging activity of* N. arbortristis *flower in different extracts. All values are expressed as the means ± SEM.

Phosphomolybdenum assay is a quantitative method to evaluate the antioxidant capacity indicated by electron donating capacity [17]. The results showed that all the extracts exhibited different degrees of activity as presented in Figure 5(a). The highest antioxidant capacity was observed in NafEEa with 30.11 ± 1.77 of AAE/100 g dw of plant material followed by NafE and NafEBu with 28.76 ± 1.51 AAE/100 g dw and 24.66 ± 2.09 AAE/100 g dw, respectively. NafEHx, NafEIn, and NafEW were found to be <5.00 ± 2.12 AAE/100 g dw which were considered to be ineffective. The phosphomolybdenum method is quantitative since the total antioxidant activity is expressed as the number of equivalents of ascorbic acid.* N. arbortristis* flower ethyl acetate, butanol, and ethanol extracts range between 30.11 and 24.66 mg AAE/g dw and other extracts were very low in concentration.

DPPH assay is a very sensitive qualitative assay for radical scavenging property and the experiment was carried out on the present study and its results can indicate the presence of antioxidant compounds in plant extracts [24].  Figure 5(b) illustrates a significant (*P* < 0.05) decrease in the concentration of DPPH due to the scavenging activities of the extract samples. The samples showed concentration dependent DPPH radical scavenging activities. The IC_50_ values of NafE, NafEHx, NafEEa, NafEBu, NafEIn, and NafEW were found to be at 32.71 ± 1.32 *μ*g/mL, 328.37 ± 2.25 *μ*g/mL, 23.98 ± 1.05 *μ*g/mL, 30.29 ± 1.78 *μ*g/mL, 104.11 ± 1.51 *μ*g/mL, and 401.15 ± 1.29 *μ*g/mL, respectively. Percentage inhibition at 40 *μ*g/mL of NafE, NafEEa, and NafEBu was found to be 52.53 ± 2.86%, 69.25 ± 3.96, and 67.98 ± 3.54%, respectively. Assessment of free radicals scavenging by DPPH method for antioxidant potential is known to be accurate, convenient, and rapid.* N. arbortristis *flowers ethyl acetate, butanol, and ethanol extracts could scavenge DPPH radical effectively 50–70%, respectively, at the highest concentration of 360 *μ*g/mL. There are reports on antioxidant property of this plant with respect to its leaves, flowers, and stem. Extensive work on leaves has been carried out but has not been much studied on flowers. Earlier report suggests that antioxidant activities from leaves, stem, and flower extracts were significantly higher in the extracts from lower polarity over the extracts from higher polarity solvent [13]. This supports our present data where the lower polarity extracts were more active than the higher polarity solvents.

### 3.3. Evaluation of Antiproliferative Activity

**Figure 6:**
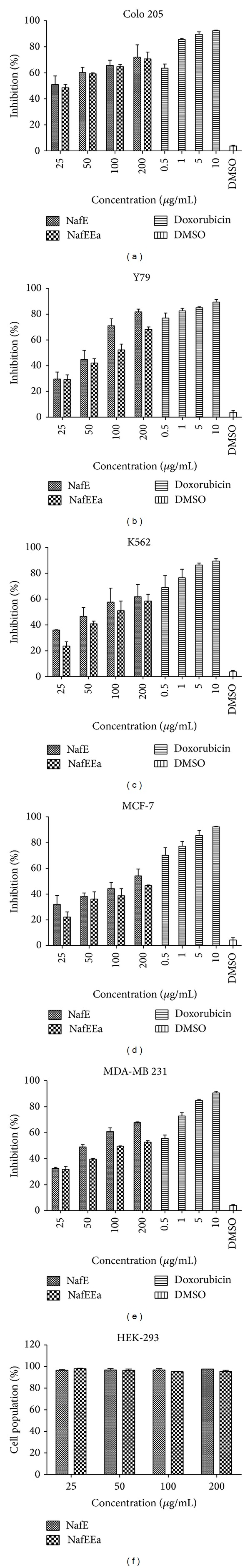
Antiproliferative activity of* N. arbortristis* flower extracts against (a) colorectal adenocarcinoma—Colo 205 cell line; (b) retinoblastoma—Y79 cell line; (c) chronic myelogenous leukemia—K562 cell line; (d) breast adenocarcinoma—MCF7 cell line; (e) breast adenocarcinoma—MDAMB231 cell line; and (f) human embryonic kidney cells—HEK cell line. Significant values (***P < 0.001, **P < 0.01, and *P < 0.05) were obtained by Student's *t*-test analysis. Composite treatments were compared using one-way analysis of variances (ANOVA) and probability values were found to be equal to or less than 0.05 for all the six cell lines.

In order to evaluate* N. arbortristis *as a potential candidate for cancer therapy, the above extracts were assayed against a panel of five different human tumor cells, colorectal adenocarcinoma (Colo 205); retinoblastoma (Y79); chronic myelogenous leukemia (K562); breast adenocarcinoma (MCF-7); breast adenocarcinoma (MDAMB-231), and the chemotherapeutic drug, Doxorubicin, as a positive control. Out of six extracts tested, only two, ethanolic extract (NafE) and ethyl acetate extract (NafEEa), were found to be active, whereas NafEHx, NafEBu, NafEIn, and NafEW extracts did not inhibit the proliferation of tumor cells, thus indicating their noncytotoxicities against the above cancer cell lines. The MTT assay that measures the formazan product at 570 nm clearly proves the cytotoxicity of the tested extracts. Figures 6(a)–6(e) show the cytotoxicity values of two active extracts in tested cell lines in comparison with normal human embryonic kidney cells (Figure 6(f)); the IC_50_values are presented in Table 2. The NafE and NafEEa were found to be cytotoxic to tested cell lines. These extracts significantly inhibited the growth of cancer cells in a concentration dependent manner as they caused significant cell death in both sensitive and resistant human cancer cell lines. NafE extract has shown the most potent cytotoxicity on all five cancer cell lines. The percentage inhibition shown by NafE was found to be in the range of 54.24 ± 3.39% to 81.81 ± 2.11% (*P* < 0.05) against all five cell lines at the highest concentration of 200 *μ*g/mL. The lowest IC_50_ value was observed against Colo 205 cell line (24.56 ± 6.63 *μ*g/mL). On the other hand, NafEEa extract at the same concentration exhibited slightly lesser percentage inhibition across the cell lines tested (46.57 ± 0.64 to 70.66 ± 5.30%; *P* < 0.05) with lowest IC_50_ values found against Colo 205 cell line (25.79 ± 2.69 *μ*g/mL). The difference in the antiproliferative effects between different extracts may have resulted from the different phytoconstituents and their concentrations contained in the extracts due to the sensitivity to the solvent used and mode of extraction. The cytotoxic effect of NafE and NafEEa was also studied in normal embryonic kidney cell line using the MTT method. The results clearly indicated that these two extracts were nontoxic and had no inhibitory effect on cell proliferation in HEK-293 and there was minimal reduction in cell survivability (Figure 6(f)). The percentage viability was above 95% at the highest concentration of 200 *μ*g/mL. This advocates that NafE and NafEEa extracts did not show any kind of toxic effect on the normal cells. Hence, the cytotoxicity of the active extracts was found to be highly selective against the cancer cell lines used.

Crocetin, carotenoid, is an active component of most ancient expensive spice, saffron (*Crocus sativus* [25]) that is also reported to possess anticancer properties [26]. In dimethylbenzanthracene (DMBA) induced skin tumorigenesis the hydroalcoholic extract of leaves of this plant at 250 mg/kg was found to be as chemopreventive [27] and 4-hydroxyhexahydrobenzofuran-7-one isolated from leaves at 20 mg/kg which inhibited the cell growth of Ehrlich ascites carcinoma cells by 43.27% and did not have any cytotoxic effect [28]. Arbortristoside A and B and iridoid glycosides are reported from seeds at 2.5 mg/kg in mice which possess anticancer activity against methylcholanthrene induced fibrosarcoma [29]. Iridoids and carotenoids are most frequent compounds identified in* N. arbortristis* and they have been reported for various biological activities [30, 31].

It was an understandable interest to know how high levels of phenolics exhibited high antioxidant activity and also influence the anticancer activity in different extracts. We expected that the extracts with the high content of the total phenolics possessing antioxidant potential possibility have high anticancer activity. Cytotoxicity screening models provide important preliminary data to help selecting plant extracts with potential antineoplastic properties for future work [32, 33]. As discussed earlier, several plant species that are rich in flavonoids are reported to prevent and possess therapeutic properties [32–35]. The flowers of this plant were reported with rich phytochemicals diterpenoids, nycanthin, flavonoids, anthocyanins, essential oil, 6, *β*-hydroxyguanine, carotenoids, *β*-monogentiobioside, *β*-digentiobioside, and various biological activities [13]. With the aid of hyphenated techniques LCMS/MS Arborside C and Crocin and *β*-monogentiobioside ester of *α*-Crocetin (8,8′-Diapocarotenedioic acid) were identified. Possibly the antioxidant and anticancer activities of ethanolic and ethyl acetate extracts of* N. arbortristis *are influenced by the presence of phytoconstituents which is in accordance with the findings of phytochemical evaluation which indicated the presence of flavonoids, phenolics, Crocin-3, and Arborside C in extracts with promising activity.

## 4. Conclusion

*N. arbortristis *is known for its varied medicinal properties in traditional ayurvedic medicine and reported for various bioactive phytoconstituents. In this study an attempt was made to investigate antiproliferative effects of different extracts of* N. arbortristis* in different human cancer cell lines apart from its antioxidant potential. The present study indicated that the ethyl acetate and ethanol extracts of* N. arbortristis *possessed the significant phenolic content and exhibited potent antioxidant and antiproliferative activities, which were comparable to the commercial antioxidant gallic acid, and the anticancer drug Doxorubicin. This seems that the* N. arbortristis *flower extracts can be considered as good sources for drug discovery. Further investigation is being carried out to identify and characterize the inherent bioactive compounds responsible for the antioxidant and anticancer activities from the ethyl acetate and ethanol extracts of* N. arbortristis*.

## Figures and Tables

**Table 1:** Phytochemical screening of flower extracts of *N. arbortristis. *

Type of extract	Phytochemical constituents						
	S	A	F	P	Sp	G	T
NafE	+	−	+	+	+	+	+
NafEHx	+	−	−	−	−	−	+
NafEEa	−	−	+	+	−	−	+
NafEBu	−	−	+	+	+	+	+
NafEIn	−	−	+	+	−	−	+
NafEW	−	−	+	+	+	+	+

Note: +ve represents presence and −ve represents absence of phytochemical; S: steroids; A: alkaloids; F: flavonoids; P: phenolics; Sp: saponins; G: glycosides; T: terpenoids.

**Table 2:** Percentage inhibition of cancer cell proliferation and IC_50_ values.

Sample	Cell type									
	Colo 205		Y79		K562		MCF		MDA-MB	
	% inhibition	IC_50_	% inhibition	IC_50_	% inhibition	IC_50_	% inhibition	IC_50_	% inhibition	IC_50_
NafE (200 *μ*g/mL)	72.01 ± 9.40	24.56 ± 6.63***	81.82 ± 2.11	55.87 ± 7.19**	61.82 ± 9.60	53.63 ± 6.84**	54.24 ± 5.39	184.36 ± 5.39*	67.80 ± 0.96	50.97 ± 3.10**
NafEEa (200 *μ*g/mL)	70.67 ± 5.30	25.79 ± 2.69***	68.06 ± 2.02	59.50 ± 3.41**	58.48 ± 5.20	98.02 ± 7.47*	46.57 ± 0.64	214.73 ± 0.64*	52.65 ± 2.02	100.81 ± 0.50*
Doxorubicin (10 *μ*g/mL)	92.33 ± 0.45	0.39 ± 0.03	89.42 ± 2.02	0.32 ± 0.10	89.42 ± 2.02	0.36 ± 0.02	92.33 ± 0.45	0.36 ± 0.09	90.65 ± 2.41	0.45 ± 0.11
DMSO 2% (Solvent Cntl)	3.71 ± 0.56	—	3.73 ± 1.53	—	3.76 ± 1.00	—	4.44 ± 1.70	—	4.02 ± 1.17	—

Values were the means of four replicates ± standard deviation (SD). Significant *P* values (***P < 0.001, **P < 0.01, and *P < 0.05) were obtained by Student's *t*-test analysis. Composite treatments were compared using one-way analysis of variances (ANOVA) and probability values were found to be equal to or less than 0.05 for all the four cell lines.
